# Cyanobacterium *Brasilonema* sp. LPS Induces Neutrophil Infiltration and Lung Inflammation in a TLR4‐Dependent Manner and Exacerbates Th2‐Dependent Lung Inflammation

**DOI:** 10.1002/iid3.70560

**Published:** 2026-10-22

**Authors:** Julie Swartzendruber, Rosalinda Monroy Del Toro, Peter Alfano, Ryan Incrocci, Michael V. Volin, Kallie Jiang, Alejandro M. S. Mayer, Philip G. Williams, Timothy J. O'Donnell, Michelle Swanson‐Mungerson

**Affiliations:** ^1^ Department of Microbiology and Immunology College of Graduate Studies, Midwestern University Downers Grove Illinois USA; ^2^ Chicago College of Osteopathic Medicine Midwestern University Downers Grove Illinois USA; ^3^ Department of Pharmacology, College of Graduate Studies Midwestern University Downers Grove Illinois USA; ^4^ Department of Chemistry University of Hawaiʽi‐Mānoa Honolulu Hawaii USA

**Keywords:** cyanobacteria, cytokines, lipopolysaccharide, lung inflammation, Th2, toll‐like receptor 4 (TLR4)

## Abstract

**Background:**

Chronic airway inflammation can be induced by both chemical and environmental factors. Airway inflammation can be induced through different immune pathways, including allergic Th2‐dependent activation of eosinophils and mast cells, Th1/Th17‐dependent neutrophil recruitment, or a combination of both mast cell and neutrophil recruitment, that is extremely difficult to treat. An environmental factor that we have shown to induce neutrophil‐mediated lung inflammation is cyanobacterium lipopolysaccharide (LPS). The study has two main objectives: first to determine whether TLR4 is required for cyanobacterium LPS‐induced lung inflammation, given our prior findings that it signals differently from other LPS types, and second to test if cyanobacterium LPS exacerbates lung inflammation in a Th2‐dependent mouse model of airway hypersensitivity.

**Methods:**

LPS from the cyanobacterium strain, *Brasilonema* sp., was purified and used for both in vivo and in vitro assays. For in vitro assays, the TLR4 inhibitor, SPA‐4, was assessed in its ability to block *Brasilonema* sp.‐dependent IL‐6 and IL‐8 induction in lung epithelial cell lines, and in vivo, the ability of *Brasilonema* sp. LPS to induce lung inflammation was assessed in TLR4‐deficient mice in comparison to controls. Finally, Th2‐driven lung inflammation was established using ovalbumin and then these mice were exposed to *Brasilonema* sp. LPS intranasally to assess if *Brasilonema* sp. LPS resulted in the exacerbation of Th2‐dependent airway inflammation.

**Results:**

Our results indicate that *Brasilonema* sp. LPS stimulates the production of cytokines both in vitro and in vivo in a TLR4‐dependent manner, since the addition of the SPA‐4 peptide blocks *Brasilonema* sp. LPS‐dependent increases in IL‐6 and IL‐8 in vitro and TLR4‐deficient mice were refractory to *Brasilonema* sp. LPS‐mediated induction of lung inflammation in vivo. Additionally, both *E. coli* and *Brasilonema* sp. LPS exacerbated airway inflammation in a Th2‐dependent model of lung inflammation. However, *Brasilonema* sp. LPS exacerbated Th2‐dependent lung inflammation without significant neutrophil infiltration.

**Conclusions:**

Despite the altered signaling through TLR4 by cyanobacterium LPS, these data demonstrate that lung inflammation induced by LPS from the cyanobacterium strain, *Brasilonema* sp., requires TLR4 and exacerbates Th2‐dependent lung inflammation. Therefore, novel interventions that target TLR4 may be beneficial in the treatment of LPS‐induced lung inflammation from environmental exposure.

## 1 Introduction

Asthma and airway inflammation in the United States and globally are a significant cause of morbidity and mortality [1, 2]. There are many causes of airway inflammation, including both chemical and environmental factors. Allergens, that are typically harmless chemicals or environmental peptides to most individuals, promote allergic airway inflammation through a predominantly Th2‐mediated immune response that results in eosinophilic lung infiltration and histamine‐induced symptoms [3]. Typically, patients with this type of allergen‐mediated asthma are treated with glucocorticoids to decrease the immune response and beta‐2‐adrenergic receptor agonizts to relax the bronchial tubes [4]. However, 10% of chronic airway inflammation is resistant to glucocorticoid treatment and accounts disproportionately for costs associated with airway inflammation treatment, due to the scarceness of other options. In these patients, they typically have a “mixed phenotype of cellular infiltrates” that include not only eosinophils, but also neutrophils [5, 6, 7]. These neutrophils exacerbate the symptoms induced by the Th2‐driven cytokines and eosinophils and promote the refractory nature of this airway inflammation to glucocorticoid treatment.

One factor that promotes neutrophil‐dependent airway inflammation and the exacerbation of Th2‐dependent airway inflammation is lipopolysaccharides (LPS) from bacteria [8, 9, 10]. LPS induces airway inflammation by stimulating the canonical LPS receptor, toll like receptor 4 (TLR4) [11]. Numerous studies, including previous studies from our laboratory, demonstrate that LPS from environmental bacteria, such as cyanobacteria, can induce neutrophil‐dependent lung inflammation with a predominant Th1/Th17 cytokine profile [12, 13, 14]. There are an estimated 8000 species of cyanobacteria that are considered gram‐negative photosynthetic prokaryotes [15] with LPS being a defining criteria. However, the LPS found among the cyanobacterial species are diverse and have been found to have varying potencies in causing human disease [16]. Therefore, it is important to assess multiple species of cyanobacteria LPS for their potencies in regard to activating the immune system. Furthermore, the ability of cyanobacteria to live in freshwater and saltwater sources or on damp surfaces increases the likelihood of cyanobacteria exposure [15, 16, 17]. For example, cyanobacteria RNA have been found in the lungs of people that live near cyanobacteria‐contaminated freshwater sources, and cyanobacterium LPS is aerosolized and inhaled by these same individuals [13, 18, 19, 20, 21]. Therefore, these data suggest that humans are exposed to cyanobacteria LPS that has the ability to impact lung function and inflammation in humans through a Th1/Th17 dependent pathway. However, the ability of cyanobacteria LPS to exacerbate Th2‐dependent airway inflammation is unknown.

The mechanisms by which allergens and LPS induce lung inflammation are through very different mechanisms. Allergens induce a Th2‐driven response which promotes the formation of Th2 cells and activation of Type 2 innate lymphoid cells (ILC2s) that produce high levels of IL‐4, IL‐5, IL‐10, and IL‐13. These cytokines promote the recruitment of eosinophils and production of allergen‐specific IgE by B cells. These IgE molecules are bound by Fcε receptors on mast cells and upon re‐exposure to the allergen, induce mast cell activation and histamine release [1, 3]. In contrast, canonical LPS‐induced lung inflammation is induced by LPS binding to toll‐like receptor 4 (TLR4) on airway epithelial cells and local immune cells. These cells produce pro‐inflammatory cytokines and chemokines that promote neutrophil and T cell recruitment to the lung to promote lung damage [22]. The T cells that are recruited during this response differentiate into Th1 or Th17 cells that are capable of continuing the pro‐inflammatory response in the lungs through the production of IFN‐gamma and IL‐17, respectively [23]. When Th2‐driven airway responses are exposed to LPS, the lungs have a “mixed infiltrate” of both neutrophils, eosinophils, and IgE‐activated mast cells, that have exacerbated pathology due to the concurrent recruitment and activation of multiple cell types. This mixed infiltrate experimental phenotype closely mimics Th2‐exacerbated lung inflammation in humans. Due to the difficulty of treating mixed infiltrate lung inflammation in humans [24, 25, 26], it is critical to understand the impact of environmental LPS independently and in combination with Th2‐induced asthma in order to identify new treatment options for these individuals.

For the current study, we set forth to ask multiple questions. Our first question was whether the ability of cyanobacteria to induce neutrophil‐dependent lung inflammation was a general property of their LPS, or if it is strain specific. We have previously published that Geitlerinema sp. LPS induces lung inflammation in vivo [12]. However, due to differences in potency among cyanobacterial species LPS, we tested the LPS from another species of cyanobacteria, *Brasilonema* sp. that is from a separate order phylogenetically in this study. *Geitlerinema* sp. is from the Oscillatoriales order while *Brasilonema* sp. is from the *Nostocales* order [17]. Therefore, we asked if *Brasilonema* sp. LPS could also induce lung inflammation and if it was through a TLR4‐dependent manner. Secondly, we asked whether *Brasilonema* sp. LPS could exacerbate Th2‐dependent lung inflammation. Our findings suggest that cyanobacterium LPS is a general activator of lung inflammation through TLR4 and can increase the severity of Th2‐dependent airway inflammation.

## 2 Materials and Methods

### 2.1 Cell Culture

A549 cell line (human lung adenocarcinoma, CCL‐185, ATCC, Manassas, VA, USA) was maintained in F‐12K media containing 10% fetal bovine serum and 1% Pen‐Strep at 37°C, 5% CO_2_.

### 2.2 Lipopolysaccharide Isolation From *Brasilonema sp*

*E. coli* LPS from strain O111:B4 was purchased from Sigma‐Aldrich (St. Louis MO, USA). LPS from the HT‐58‐2 strain of *Brasilonema* sp [27]. was purified as described previously [28, 29]. Briefly, the harvested freeze‐dried biomass was extracted in accordance with the commonly utilized hot phenol‐water extraction [30, 31]. Briefly, up to 1 g of biomass was transferred to a 50 mL centrifuge tube and incubated in 40 mL of 1:1, distilled H_2_O: 90% aq. phenol in a 68°C water bath for 30 min. After this time, the mixture was vigorously vortexed and subsequently centrifuged at 2500 r.c.f. for 15 min. The aqueous supernatant of the biphasic mixture was collected carefully so as not to remove cellular debris. This procedure was repeated twice more by adding fresh volumes of distilled H_2_O to the extracting centrifuge tube. The pooled extracts were dialyzed overnight in one liter of Milli‐Q H_2_O in a 3000 Da MWCO dialysis membrane in order to remove residual phenol. This dialysis was repeated a total of three times. The dialyzed extract was freeze‐dried. At this stage, the crude LPS was resuspended to a concentration of 5 mg/mL in 0.1 M TRIS‐HCl buffer and incubated at room temperature overnight while on an orbital shaker in the presence of 25 μg/mL of RNase. The material was then dialyzed as described previously, freeze‐dried and stored at –20°C for later chemical analysis.

### 2.3 Purity Assessment and Activity of *E. coli* and *Brasilonema* sp. LPS

To assess the nucleic acid content of the lipopolysaccharide sample, UV measurements were taken on a BioSpec‐nano spectrophotometer. The measured readings at 230, 260, and 280 nm of a 100 μg/mL aqueous solution of purified LPS were used to calculate the mass percentage of dsDNA or RNA. Results indicated a concentration of dsDNA (1.1 ng/ul) and RNA (0.8 ng/ul). We further analyzed the purity of the LPS using SDS‐PAGE gel. Briefly, the Molecular Probes Pro‐Q Emerald 300 Lipopolysaccharide Gel Stain Kit from Thermofisher (Waltham MA, USA), was used to visualize the LPS samples after SDS polyacrylamide gel electrophoresis per the manufacturer's instructions and the data are shown in Supporting Information Figure 1A. Finally, we demonstrated the activity of our *Brasilonema* sp. LPS relative to our *E. coli* LPS control using the Limulus Amebocyte Lysate (LAL) endotoxin assay kit from Genscript (Piscataway NJ, USA), which demonstrated significant activity (Supporting Information Figure 1B).

### 2.4 In Vivo LPS exposure

All animal experiments were reviewed and approved by the Institutional Animal Care and Use Committee at Midwestern University (Approval #3277). C57Bl/6 mice were housed in the American Association of Laboratory Animal Care (AALAC)‐accredited Animal Research Facility at Midwestern University (Downers Grove, IL, USA). All mice were housed in temperature‐controlled (21.2°C) rooms under a 12 h light/dark cycle and provided with autoclaved food and deionized water ad libitum. C57Bl/6 mice or TLR4‐deficient (strain: 029015 B6(Cg)‐Tlr4/J) mice were purchased from Jackson Laboratories, (Bar Harbor, ME, USA) and bred in house under standard breeding protocols. At 4–8‐weeks of age, both male and female C57Bl/6 mice or TLR4‐deficient mice were equally distributed and randomly assigned to treatment groups. Assigned mice were treated intranasally with 1 µg/g of *E. coli* LPS (Sigma‐Aldrich, St. Louis, MO, USA), 1 µg/g of *Brasilonema* LPS, or 50 µl of PBS alone after exposure to 3% isoflurane as an anesthetic. After 24 h, mice were euthanized via inhalation of CO_2_ by gradual inflow method, followed by the collection of bronchial alveolar lavage (BAL) by flushing lungs with 1 ml BAL fluid (10% FCS, 1 mM EDTA, 1X PBS). Lungs were excised and fixed in formalin for 24 h. BAL fluid (BALF) was counted and cytospun onto slides and analyzed for neutrophils, lymphocytes, eosinophils, and macrophages using Diff‐Quik staining (Dade Behring, Deerfield, IL, USA) as described previously [12, 32].

### 2.5 In Vivo Exacerbation Model

At 4‐8 weeks of age, C57Bl/6 mice were intraperitoneally sensitized with 10 μg or 50 μg of Ovalbumin (OVA) (Worthington‐Biochem, Lakewood, NJ, USA) adsorbed to 3 mg Alum‐adjuvant (Thermo Fisher, Waltham, MA, USA) as described previously [33]. On Days 13–16, the mice were intranasally challenged with 10 mg/ml or 0.1 mg/ml of Ovalbumin. Subsequently, the mice were intranasally challenged with 50 ng/ml of either *E. coli* LPS, *Brasilonema* LPS (2.5 ng LPS per mouse), or 50 µl of PBS alone on days 22–24. 24 h after the final LPS exposure, the mice were harvested and BAL, serum and lungs were collected for analysis and blinded for analysis in order to not skew the interpretation of the data. For example, pathological scoring was done by a pathologist who only had access to a number for the sample, not the group from which the sample was derived.

### 2.6 Flow Cytometry

Cells collected from BALF were washed in FACS buffer (1x PBS/1% FCS) and blocked with TruStain FcX anti‐mouse CD16/32 (Biolegend, San Diego, CA, USA) for 30 min. Cells were washed again in FACS buffer and stained with PE anti‐mouse CD11b, APC/Fire750 anti‐mouse F4/80, FITC anti‐mouse SiglecF, and Brilliant Violet 510 anti‐mouse Ly‐6G (Biolegend, San Diego, CA, USA) for 30 min in the dark on ice. The cells were then washed and measured using CytoFLEX flow cytometer. Cells from the BALF were gated on CD11b^+^ and neutrophils were subsequently quantified as CD11b^+^Ly‐6G^+^Siglec F^‐^, while eosinophils were designated as CD11b^+^Ly‐6G^−^ Siglec F^+^ F4/80^−^. The gating strategy for our flow cytometry is exemplified in Supporting Information Figures 2–3.

### 2.7 Multiplex

Cell supernatants from BALF were plated for analysis using the MILLIPLEX Multiplex Assay (Millipore Sigma, Burlington, MA, USA) according to manufacturer's protocol. The following analytes were measured: IFN‐gamma, IL‐6, IL‐10, IL‐17a, KC (IL‐8), MIP‐2, RANTES, and TNF‐α. Results were gathered using the MAGPIX plate reader (Millipore Sigma, Burlington, MA, USA) and data analysis was performed using the MILLIPLEX Analyst Software (Millipore Sigma, Burlington, MA, USA).

### 2.8 Hematoxylin and Eosin Staining and Lung Histopathology Scoring

Formalin‐fixed lung tissues were embedded in paraffin wax and cut into 5 µm sections. Slides were deparaffinized in xylene for 15 min, rehydrated by transferring through graded alcohols and incubating in 3% hydrogen peroxide for 10 min, then incubating in hematoxylin (VWR, Radnor, PA, USA) for 2 min. Slides were washed and dipped in ammonia water until sections turned blue. Slides were rinsed and dipped in eosin (VWR, Radnor, PA, USA) 20x followed by dehydration through graded alcohols, incubated in xylene for 1 min and sealed. Sections were semi‐quantitatively scored using the methods described previously [34, 35] by a blinded pathologist. Briefly, lung inflammation was graded based on the following criteria on a scale from 0 to 4: level of perivascular edema, severity and extent of peri‐bronchial/bronchiolar infiltrates, goblet cell metaplasia, and eosinophilic macrophages in alveolar spaces. The sum of each individual assessment was used to generate the overall score for pulmonary inflammation.

### 2.9 ELISA

A549 (7.5 × 10^4^) were seeded in a 96‐well plate and incubated for 24 h. Cells were treated with *Brasilonema* sp. LPS (1 × 10^5^ ng/ml) and/or various concentrations of the SPA‐4 peptide (Focus Biomolecules, Plymouth Meeting, PA, USA) for 24 h. Supernatants were collected and plated for IL‐6 or IL‐8 production using LEGEND MAX human IL‐6 or IL‐8 ELISA kit (Biolegend, San Diego, CA, USA), respectively, according to manufacturer's instructions. Results were obtained using a Multiskan FC Microplate Photometer (Thermo Fisher, Waltham, MA, USA).

### 2.10 Statistics

Each experiment was performed at least 3 times. Experiments were first analyzed by an ANOVA (either one‐way ANOVA or two‐way ANOVA, when appropriate) followed by a Tukey post‐hoc test. All analysis was done using GraphPad Prism 9 Software (La Jolla, CA, USA).

## 3 Results

Since cyanobacteria LPS is aerosolized near fresh water sources [13, 21, 36] and our laboratory previously demonstrated that LPS from a specific species of cyanobacteria, *Geitlerinema* sp., induces lung inflammation in vivo [12], we wanted to test if this was a common phenomenon beyond just this species. Therefore, we tested if LPS from another cyanobacterium sp., *Brasilonema* sp. HT‐58‐2, induces lung inflammation in vivo by exposing C57Bl/6 mice intranasally to *Brasilonema* sp. LPS and assessing lung inflammation and neutrophil recruitment 24 h later. For these experiments, *E. coli* LPS was used as a positive control for inducing lung inflammation as previously described [12]. As shown in Figure 1A, hematoxylin and eosin staining of lung tissue 24 h after LPS exposure demonstrated inflammation in the lungs of mice exposed to either *E. coli* or *Brasilonema* sp. LPS. When the bronchoalveolar fluid (BALF) was analyzed by flow cytometry, the data indicate that LPS from both *E. coli* and *Brasilonema* sp. LPS induces CD11b^+^Ly‐6g^+^SiglecF^‐^ neutrophil infiltration (Figure 1B left side). To confirm and expand these findings, we also used Diff‐Quik staining to allow for the analysis of multiple cell types within the BALF. When this technique was used, the results indicated that both *E. coli* and *Brasilonema* sp. LPS increased neutrophil numbers with diminished numbers of macrophages (Figure 1B right side), which is consistent with previous LPS‐induced lung inflammation [12]. Finally, we analyzed the levels of both chemokines and cytokines in the BALF of *Brasilonema* sp. LPS‐treated mice to assess if *Brasilonema* sp. LPS induced a pro‐inflammatory environment in the lungs. As shown in Figure 1C, *Brasilonema* sp. LPS enhanced the production of the pro‐inflammatory cytokine IL‐6 and the chemokine KC (IL‐8), while not promoting the production of anti‐inflammatory cytokines such as IL‐10 (Supporting Information Figure 4). Taken together, these data suggest that *Brasilonema* sp. LPS promotes lung inflammation in a similar manner to other cyanobacteria species, such as *Geitlerinema* sp. LPS [12].

**Figure 1:**
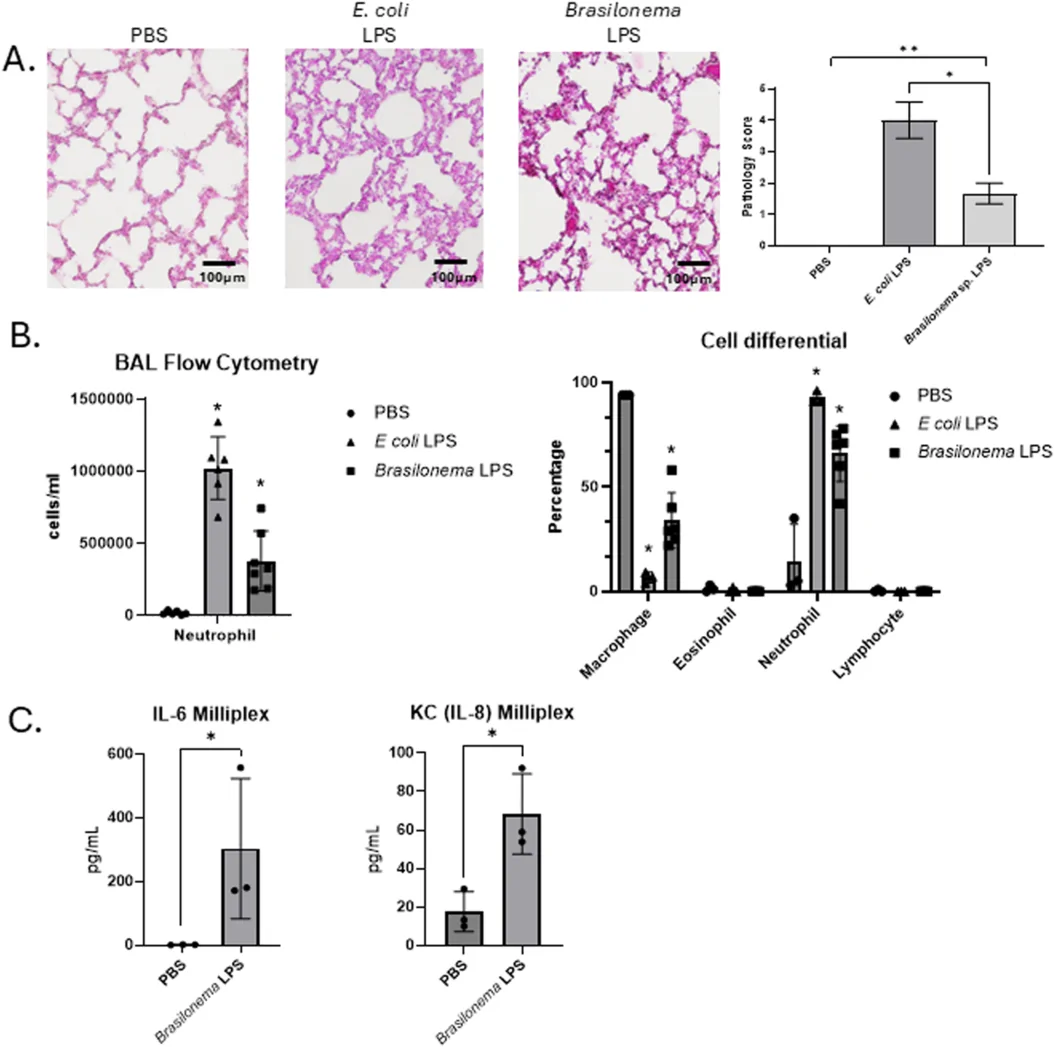
*Brasilonema* sp. LPS induces lung inflammation. (A–C) Mice were exposed to either PBS or LPS for 24 h before lung tissue and BALF were collected. (A) Hematoxylin and Eosin staining of lung tissue. The bar graph represents the pathology score combined from 3 mice per group as described in the Methods section. * indicates *p* < 0.05 between the pathology score comparing lungs from mice exposed to either *E. coli* and *Brasilonema* sp. LPS, ** indicates a *p* < 0.05 for both groups when compared mice only exposed to PBS. (B) Analysis of BALF cellular infiltration by either Flow cytometry (left side) or Diff‐Quik staining (right side) (C) Milliplex analysis of pro‐inflammatory cytokine levels in the BALF. Data are either representative of 6 mice with similar results (A) or a combination of 3–6 mice (B–C). Each dot represents an individual mouse * indicates *p* < 0.05 when compared to mice exposed to only PBS.

Previous studies indicate that lipopolysaccharides from Gram‐negative bacteria exacerbate classical Th2‐driven lung inflammation in both animals and humans [8, 9, 10]. To mimic humans with Th2‐dependent airway inflammation that are exposed to cyanobacteria LPS, we used an established mouse model of LPS‐driven allergic airway exacerbation [33]. For these studies, mice were sensitized to ovalbumin (OVA) on day zero with Th‐2‐driven airway inflammation and induced on days 13–16 with intranasal application of OVA. On days 19–21, the mice were exposed to low concentrations of either *E. coli* LPS or *Brasilonema* sp. LPS and then lung histology and BALF were analyzed 24 h after the final LPS exposure (Figure 2A). When the lungs were assessed by hematoxylin and eosin staining for lung inflammation, the OVA‐only exposed animals displayed increased lung inflammation in comparison to PBS controls (Figure 2B left column) and elevated eosinophil infiltration (Figure 2C) as expected. When the lungs were exposed to OVA and *E. coli* LPS, the lungs showed an increase in lung inflammation, as previously published (Figure 2B lower center column) and an increase in both eosinophils from the OVA and neutrophils from the *E. coli* LPS (Figure 2C). Interestingly, when the pathology was scored from mice exposed to both OVA and *Brasilonema* sp. LPS, the lungs showed an increased level of inflammation when compared to OVA alone by hematoxylin and eosin staining (Figure 2B) and eosinophils infiltration without the additional recruitment of neutrophils as determined by flow cytometry (neutrophils: CD11b^+^Ly‐6G^+^Siglec F^‐^ and eosinophils: CD11b^+^Ly‐6G^−^ SiglecF^+^ F4/80^−^) (Figure 2C). While this finding was surprising, the pathology scoring (which assesses edema and cellular infiltration in the lung tissue) identified inflammation induced by the *E. coli* and *Brasilonema* sp. LPS alone without consistent and significant neutrophil infiltration (Figure 2C). These findings suggest that low levels of *E. coli* and *Brasilonema* sp. LPS over multiple days promote some level of lung pathology independent of neutrophil infiltration when extremely low levels of *E. coli* and *Brasilonema* sp. LPS are used (50 ng used in Figure 2 vs. 1 ug/ml in Figures 1 and 5).

**Figure 2:**
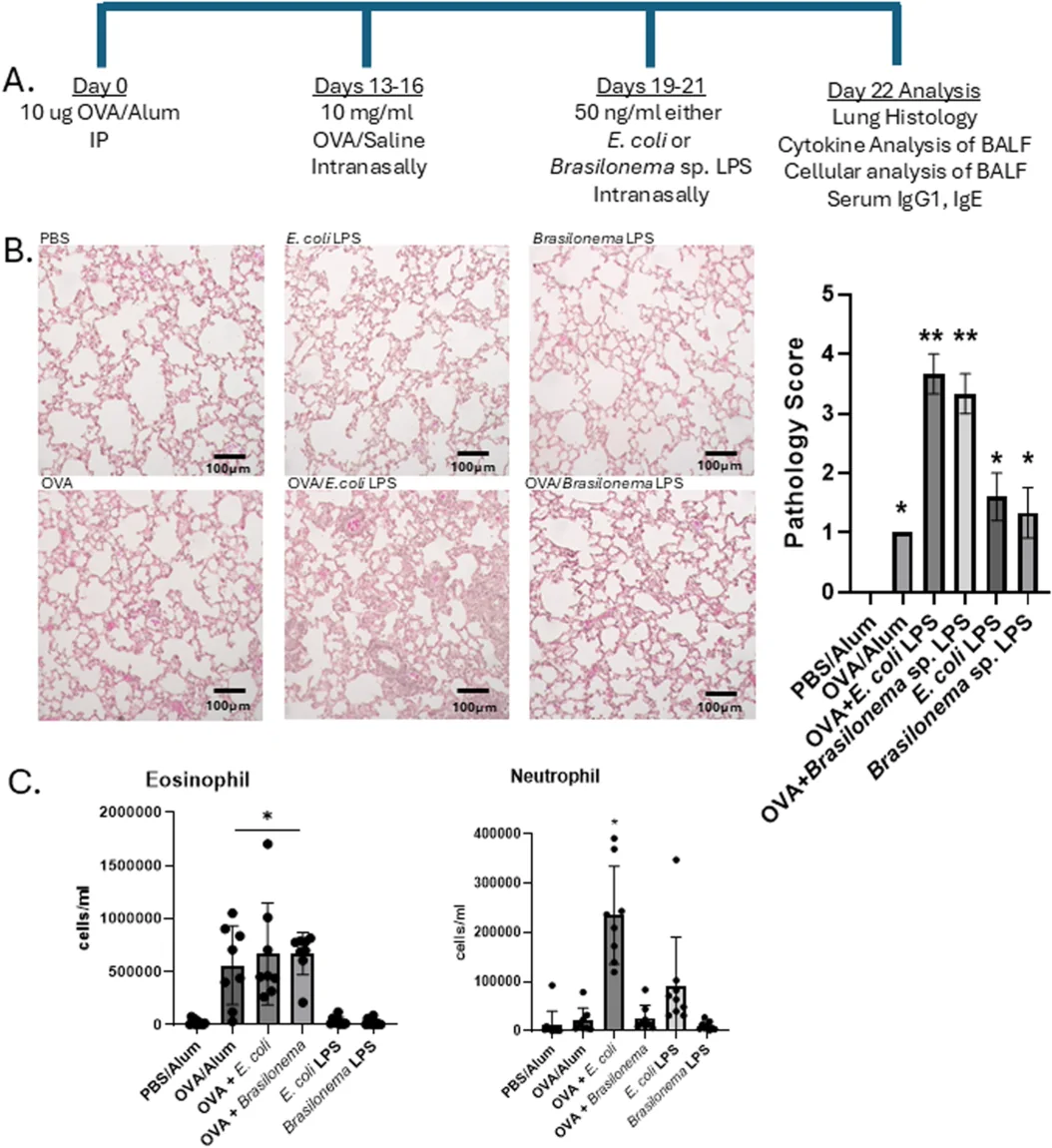
*E. coli* and *Brasilonema* sp. LPS exacerbate OVA‐mediated lung inflammation with high OVA sensitization concentrations. (A) Experimental design (B) Hematoxylin and Eosin staining of lung tissue on day 22 including the pathology score combined from 3 to 6 mice per group as described in the Methods section. * indicates *p* < 0.05 comparing the pathology score comparing lungs from mice exposed to PBS only ** indicates *p* < 0.05 comparing groups significantly different from OVA/Alum alone (C) Analysis of BALF cellular infiltration by flow cytometry. Data are either representative of 3 mice (B) or a combination of 3 mice (C) Each dot represents an individual mouse * indicates *p* < 0.05 when compared to mice exposed to only PBS/Alum.

Since *Brasilonema* sp. LPS enhanced lung inflammation and eosinophil infiltration without neutrophils under these conditions, we wanted to determine whether the low level of *Brasilonema* sp. LPS exposure influences the cytokine microenvironment in the BALF. To assess this possibility, we measured cytokine levels of the BALF 24 h after final LPS exposure and these results are shown in Figure 3. The OVA‐model induced IL‐4 production in all OVA‐exposed mice, as expected (Figure 3A). Concurrently, the levels of OVA‐specific IgG1 and IgE that are promoted by IL‐4 were increased across all OVA‐sensitized groups (Figure 3B). However, as shown in Figure 3A, the addition of *E. coli* LPS to OVA‐sensitized mice also increased the presence of Th1/Th17 cytokines (IFN‐gamma and IL‐17) that are associated with a “mixed infiltrate” of neutrophils and eosinophils as seen in Figure 2 for these conditions. Interestingly, *Brasilonema* sp. LPS did not influence Th1/Th17 cytokines, but it was able to increase the amount of TNF‐α and RANTES in the BALF of OVA‐sensitized mice (Figure 3A) suggesting that these low levels of *Brasilonema* sp. LPS can promote low level inflammation in the lungs of these mice without the recruitment of neutrophils.

**Figure 3:**
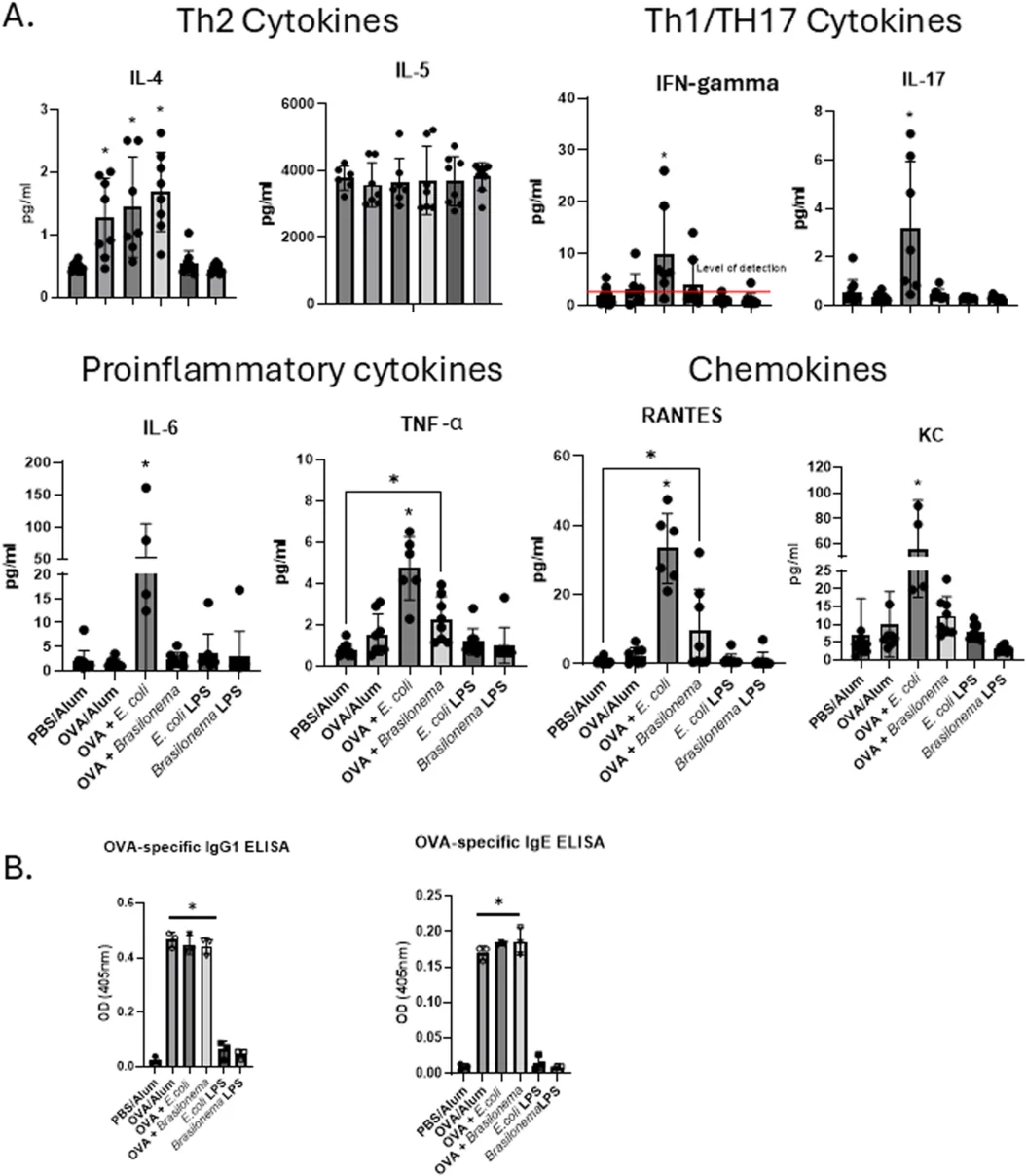
E. *coli* but not *Brasilonema* sp. LPS induces cytokines that promote a “mixed phenotype” of cellular infiltration in OVA‐mediated lung inflammation with high OVA sensitization concentrations. (A) BALF 24 h after LPS exposure was assessed using a Milliplex as described in the Methods. The red line indicates the threshold of detection for IFN‐gamma (B) Analysis of BALF IgG1 and IgE levels 24 h after LPS exposures. Data are a combination of either 8 mice (A) or 3 mice (B) Each dot represents an individual mouse * indicates *p* < 0.05.

For these initial experiments assessing the impact of *Brasilonema* sp. LPS on Th2‐driven airway inflammation, we used a high concentration of OVA to induce Th2‐driven airway inflammation in the mice as was previously described [33]. It was possible that this high concentration of OVA may have generated such a Th2‐dominant phenotype that the low dose of *Brasilonema* sp. LPS was unable to skew the response away from a focused Th2 response to a mixed‐infiltrate response. To test this possibility, we repeated these studies with 0.1–mg/ml OVA to induce airway inflammation on days 13–16 instead of 10 mg/ml on days 13–16 (as was done for Figure 2). As shown in Figure 4, flow cytometry data indicate that even with a lower dose of sensitizing OVA, the lungs at day 22 demonstrated eosinophilic infiltration in all OVA‐sensitized groups (Figure 4C), while neutrophil exacerbation was only seen in mice exposed to *E. coli* LPS (either in the presence or absence of OVA) Figure 4C. These data indicate that even under lower Th2‐promoting conditions of airway inflammation, *Brasilonema* sp. LPS exacerbates lung inflammation without inducing neutrophil infiltration and a mixed cell infiltrate.

**Figure 4:**
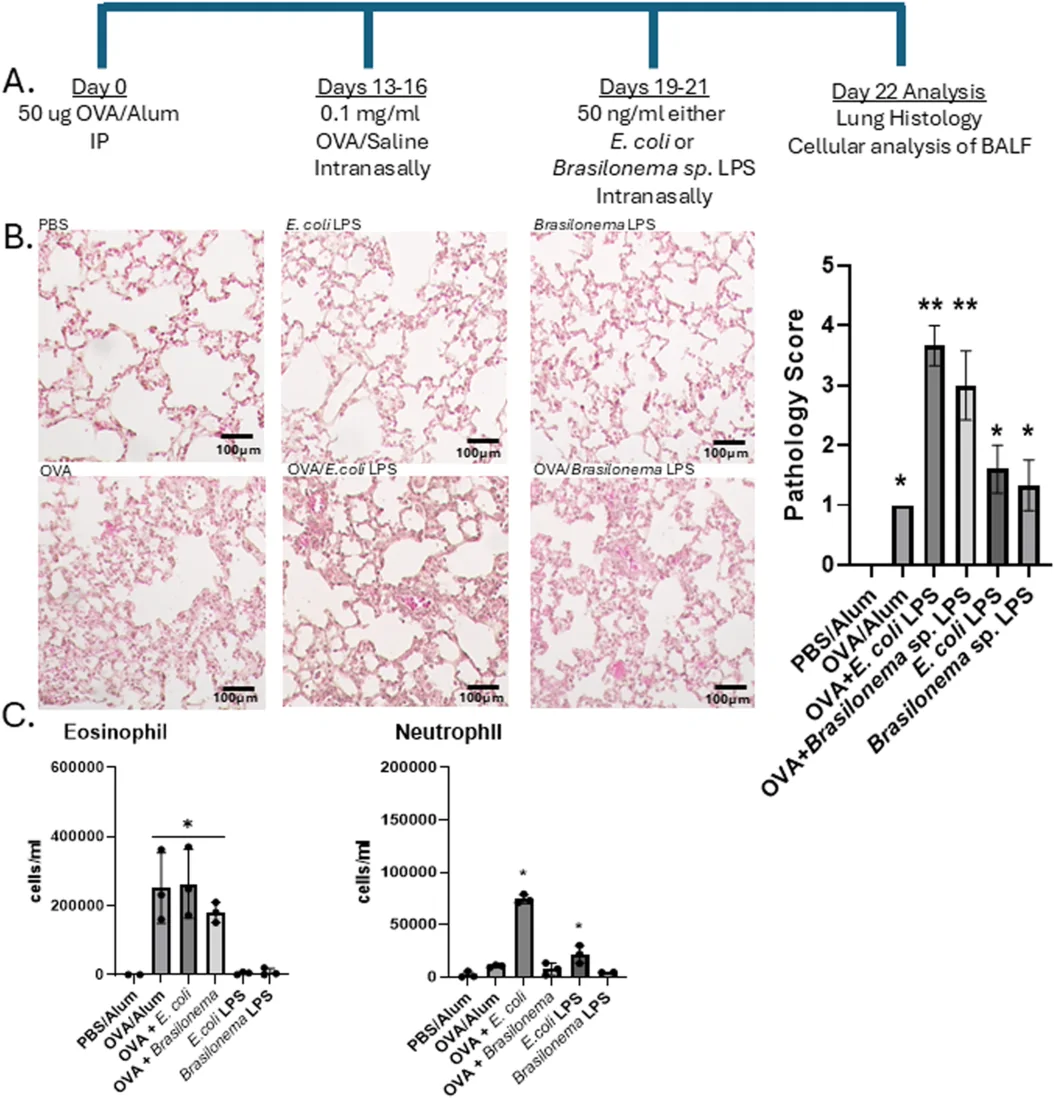
E. *coli* and *Brasilonema* sp. LPS exacerbate OVA‐mediated lung inflammation with low OVA sensitization concentrations. (A) Experimental design (B) Hematoxylin and Eosin staining of lung tissue on day 22 including the pathology score combined from 3 to 6 mice per group as described in the Methods section * indicates *p* < 0.05 comparing the pathology score comparing lungs from mice exposed to PBS only ** indicates *p* < 0.05 comparing groups significantly different from OVA/Alum alone (C) Analysis of BALF cellular infiltration by flow cytometry. Data are either representative of 3 mice (B) or a combination of 3 mice (C) Each dot represents an individual mouse * indicates *p* < 0.05 when compared to mice exposed to only PBS/Alum.

Our results demonstrate that *Brasilonema* sp. LPS exacerbates Th2‐dependent lung inflammation without significantly inducing neutrophil recruitment (Figures 2 and 4). Similarly, we found that *Brasilonema* sp. LPS alone never reached the same extent of inflammation and/or neutrophil infiltration as that of *E. coli* LPS (Figure 1A–B), which is similar to what we had seen using *Geitlerinema* sp. LPS [12, 28, 29]. Additionally, our previous data suggested that *Geitlerinema* sp. LPS induced distinct signaling through the predominant LPS receptor, Toll‐like receptor‐4 (TLR4) [28], likely due to compositional differences in sugar moieties in cyanobacterium LPS [29]. Therefore, it was possible that the reason both *Brasilonema* sp. LPS and *Geitlerinema* sp. LPS induced a reduced and/or slightly altered response to cyanobacteria LPS was due to alternate receptor usage instead of TLR4. To test this possibility, we initially switched to an in vitro assay to assess whether *Brasilonema* sp. LPS induced the production of IL‐6 (a pro‐inflammatory cytokine) and IL‐8 (a chemokine that recruits neutrophils) by airway epithelial cells, and if the TLR4‐specific antagonist, SPA‐4, blocked this interleukin induction. To achieve this, the airway epithelial cell line, A549, was exposed to *Brasilonema* sp. LPS in the absence or presence of increasing concentrations of SPA‐4 for 24 h and IL‐6 and IL‐8 levels were determined by ELISA. As shown in Figure 5, *Brasilonema* sp. LPS significantly increased both IL‐6 and IL‐8 levels and that the addition of SPA‐4 peptide significantly blocked the induction of both IL‐6 (Figure 5A) and IL‐8 (Figure 5B) in a concentration‐dependent manner without significantly impacting cell viability at 1 and 10 uM with some impact at 100 uM (Supplemental Figure 5). We subsequently confirmed if *Brasilonema* sp. LPS utilizes TLR4 in vivo to mediate lung inflammation. For these experiments, both C57Bl/6 and TLR4‐deficient (TLR4–/–) mice were exposed to either PBS, *E. coli* LPS, or *Brasilonema* sp. LPS for 24 h before lung inflammation was analyzed. Wildtype C57Bl/6 mice exposed to either *E. coli* or *Brasilonema* sp. LPS demonstrated lung inflammation and pathology in comparison to the PBS control mice, which was absent in TLR4−/− mice exposed to either form of LPS (Figure 5C). When the BALF was analyzed for the infiltration of neutrophils associated with LPS‐induced lung inflammation as seen in Figure 1, the BALF from TLR4−/− mice exposed to *Brasilonema* sp. LPS did not have any infiltrating neutrophils by flow cytometry (Figure 5D left side) or Diff‐Quik analysis (Figure 5D right side), while there was still some residual neutrophil lung infiltration in the TLR4−/− BALF fluid exposed to *E. coli* LPS. When BALF was analyzed for the pro‐inflammatory cytokine IL‐6 and the chemokine KC (IL‐8), both remain at baseline levels in TLR4 deficient mice after LPS exposure (Figure 5E) in comparison to wildtype mice exposed to *Brasilonema* sp. LPS. All other cytokines analyzed were unchanged in both wildtype and TLR4‐deficient mice (Supporting Information Figure 6). Taken together, these data suggest that *Brasilonema* sp. LPS requires TLR4 to mediate lung inflammation and promote a pro‐inflammatory cytokine environment in vivo.

**Figure 5:**
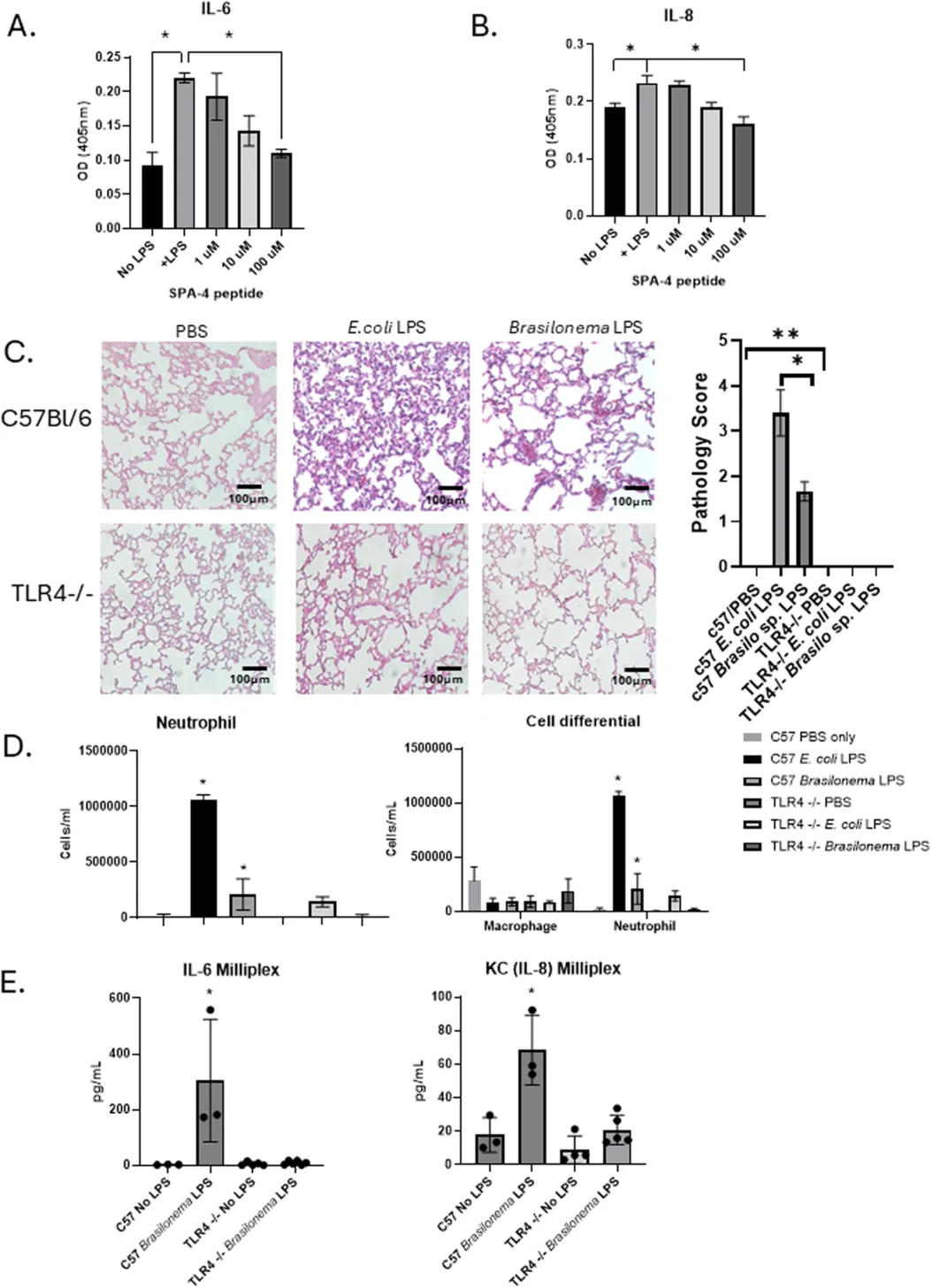
*Brasilonema* sp. LPS requires TLR4 to influence cytokine production and lung inflammation. (A, B) A549 lung epithelial cell line were exposed to *Brasilonema* sp. LPS in the absence or presence of the TLR4 antagonist (SPA‐4) for 24 h before IL‐6 (A) or IL‐8 (B) by ELISA (C, D) Wildtype and TLR4‐deficient mice were exposed to either PBS or LPS for 24 h before lung tissue and BALF were collected. (C) Hematoxylin and Eosin staining of lung tissue including the pathology score combined from 3 to 6 mice per group as described in the Methods section. * indicates *p* < 0.05 between the pathology score comparing lungs from mice exposed to either *E. coli* and *Brasilonema* sp. LPS, ** indicates a *p* < 0.05 for both groups when compared mice only exposed to PBS. (D) Analysis of BALF cellular infiltration by either Flow cytometry (left side) or Diff‐Quik staining (E) Milliplex analysis of pro‐inflammatory cytokine levels in the BALF Each dot represents an individual mouse * indicates *p* < 0.05 when compared to mice exposed to only PBS.

## 4 Discussion

Previous data from our laboratory demonstrated that there were compositional differences between cyanobacterium LPS and *E. coli* LPS, which is known to stimulate TLR4 [29]. We further demonstrated that cyanobacterium LPS exposure led to both NF‐kB and p38 activation, but did not result in IRF3 phosphorylation, which suggested that cyanobacteria induced altered signaling through TLR4 [28]. However, we did not specifically test whether cyanobacterium LPS was stimulating TLR4 to induce signal transduction and/or cellular activation. While TLR4 is the predominant LPS receptor on human immune cells [37], multiple studies indicate that LPS is recognized by transient receptor potential (TRP) cation channels on airway epithelial cells [38] and/or intracellular caspases to promote immune cell activation and pro‐inflammatory cytokine production [37, 39]. Therefore, we tested the requirement for TLR4 and identified that the cyanobacterium LPS, *Brasilonema* sp. LPS, required TLR4 in murine in vivo lung inflammation models and on human airway epithelial cells (Figure 5).

Additionally, *Brasilonema* sp. LPS exacerbated Th2‐mediated lung inflammation in a manner similar to *E. coli* LPS but apparently through different mechanisms (Figures 2, 3, 4) as evidenced by a lack of neutrophil infiltration with *Brasilonema* sp. LPS. While these data are quite convincing, there are multiple reasons for which there are different outcomes between *Brasilonema* sp. and *E. coli* LPS. First, the concentration of *Brasilonema* sp. LPS used in the established exacerbation model (Figures 2, 3, 4) is significantly lower than the concentration of *Brasilonema* sp. LPS that induced neutrophil accumulation alone (Figures 1 and 5). Since we have consistently demonstrated that cyanobacterium LPS is a weaker stimulus for immune cell activation [28, 29] and lung inflammation [12], it is possible that if the concentration of *Brasilonema* sp. LPS was increased during the exacerbation phase of this experimental design, *Brasilonema* sp. LPS would induce neutrophil recruitment in addition to the pathology seen in the Th2‐dependent eosinophilic model. This idea is supported by the fact that in Figures 3 and 5, mice only exposed to low levels of *Brasilonema* sp. LPS did not demonstrate neutrophil infiltration in the BALF, while mice exposed only to low levels of *E. coli* LPS did have neutrophil infiltration in the BALF. Therefore, initiating experiments that expose Th2‐sensitized mice to higher *Brasilonema* sp. LPS may demonstrate both neutrophil infiltration and pathology. We did not perform these experiments, since using a high dose of *E. coli* LPS during the exacerbation phase that was equivalent to the level of *Brasilonema* sp. LPS that induces neutrophil infiltration would be lethal to the mice. However, others have reported that cyanobacterium LPS from freshwater cyanobacterium blooms are as potent as *E. coli* LPS [40, 41, 42] and this is another possible future avenue to determine if cyanobacteria LPS isolated from freshwater blooms are more potent in our model and could exacerbate Th2‐dependent lung inflammation.

Additionally, we are using an exacerbation model that is limited to 3 days of *Brasilonema* sp. LPS exposure. In humans, it is likely that individuals are chronically exposed to cyanobacterium‐tainted water and aerosols [43, 44, 45]. Therefore, it is still possible that cyanobacterium LPS could exacerbate Th2‐driven lung inflammation in humans under these conditions generating a “mixed cellular infiltrate” lung inflammation that is significantly more challenging to treat than a Th2‐dependent response. Evidence for *Brasilonema* sp. LPS influencing the inflammatory status of the lung was found with elevated levels of TNF‐α and RANTES in OVA‐sensitized mice treated with *Brasilonema* sp. LPS, in addition to increased pathology score. It is possible that chronic production of these cytokines from repeated cyanobacterium LPS exposure would ultimately produce a pro‐inflammatory environment that would result in neutrophilic airway inflammation. Future experiments are planned to address these possibilities.

Interestingly, the mechanism by which *Brasilonema* sp. LPS exacerbates Th2‐dependent airway inflammation is more limited than that seen with *E. coli* LPS. *E. coli* exacerbates lung inflammation with accompanying neutrophil recruitment, while *Brasilonema* sp. LPS exacerbates Th2‐dependent lung inflammation without neutrophil recruitment. However, as shown in both Figures 2 and 4, both *E. coli* and *Brasilonema* sp. LPS alone induced lung pathology independently of significant neutrophil recruitment. This suggests that LPS stimulation of lung tissue is sufficient to induce low level pathology. In support of this conclusion, previous studies have demonstrated that LPS‐mediated stimulation of endothelial cells contributes to lung pathology in the LPS model of airway inflammation [46]. This endothelial activation can promote edema and changes in adhesion molecule expression that can influence the retainment of cells in the tissue that may be missed in the BALF [47]. This idea is supported by the fact that we show that *Brasilonema* sp. LPS specifically stimulates A549 (pulmonary carcinoma cells) to produce IL‐6 (Figure 5A), which is a significant promoter of vascular permeability that results in airway edema [48]. Therefore, we hypothesize that the neutrophil‐independent pathology seen in the *Brasilonema* sp. LPS exacerbation of Th2‐driven airway inflammation is through stimulation of TLR4 receptors within the lung that induce localized edema and cellular retention that is scored in that pathology scoring mechanism utilized for these studies.

Finally, our data indicate that the LPS from multiple cyanobacteria species is capable of inducing lung inflammation (current data and [12]), suggesting that this may be a general phenomenon. While there are limited cyanobacteria LPS that can block the ability of *E. coli* LPS from stimulating TLR4 and are considered TLR4 antagonists in this context [49], other researchers continue to add to this body of literature indicating the cyanobacterium LPS purified from cyanobacterium blooms is highly immunomodulatory. Cyanobacteria LPS has been shown to activate human lung carcinoma cells (Figure 5), monocytes/macrophages [29, 42], peripheral blood mononuclear cells [41], keratinocytes [40, 41], intestinal epithelial cells [41], murine B cells [28], rat microglia [50], and even fish intestinal cells [51]. These broad implications suggest that cyanobacterium blooms, which are increasing due to global warming and other environmental factors [21], will continue to have significant impacts on both human and animal populations. While many of these previous studies utilized in vitro techniques, continuing to address the impact of cyanobacterium LPS using in vivo models of disease will directly contribute to our understanding of the impact of cyanobacteria on human and animal health. In light of the fact that TLR‐4 antagonists such as SPA‐4 used in this study have been found by other groups to be well tolerated in animal studies [34] and can block *E. coli* LPS activity in vivo with minimal toxicity [52], our data suggests that in vivo data such as these may lead to a better understanding of lung inflammation near cyanobacterial blooms and novel protocols to treat patients. Additionally, these types of studies hopefully will continue to raise awareness to the harms of cyanobacterium blooms and the need to monitor and treat our water systems effectively.

## Author Contributions

**Julie Swartzendruber:** conceptualization, methodology, writing – review and editing, formal analysis. **Rosalinda Monroy Del Toro:** methodology, validation, investigation, formal analysis, writing – review and editing. **Peter Alfano:** investigation, methodology, validation, writing – review and editing, formal analysis. **Ryan Incrocci:** investigation, methodology, validation, writing – review and editing, formal analysis. **Michael V. Volin:** validation, formal analysis, methodology. **Kallie Jiang:** investigation, methodology, validation, writing – review and editing. **Alejandro M. S. Mayer:** conceptualization, writing – review and editing. **Philip G. Williams:** conceptualization, writing – review and editing. **Timothy J. O'Donnell:** validation, writing – review and editing. **Michelle Swanson‐Mungerson:** conceptualization, investigation, funding acquisition, writing – original draft, methodology, validation, formal analysis, project administration, supervision.

## Ethics Statement

All animal experiments were reviewed and approved by the Institutional Animal Care and Use Committee at Midwestern University (Approval #3277).

## Conflicts of Interest

The authors have no conflict of interests to declare.

## Supporting information


**Figure S1:** Purity and Endotoxin LPS Activity of Brasilonema sp. and *E. coli* sp. LPS. (A) Purified Brasilonema sp. LPS (lanes 4–5) was tested for purity and compared to the *E. coli* LPS standard (lane 2). The data demonstrate no major impurities. (B) The LAL Endotoxin (LPS) assay was used according to manufacturer instruction and data are demonstrated as active EU (Endotoxin Units) per ug relative to the blank and commercially provided positive control.

**Figure S2:** Representative flow cytometry plots and gating to identify eosinophils for Figures 2–4. Eosinophils were CD11bhiSiglecF+Ly6G‐F4/80‐. A. Eosinophils were identified initially by CD11b and SiglecF expression. These cells were confirmed to be Ly6G‐ (B) and F4/80‐ (C).

**Figure S3:** Representative flow cytometry plots and gating for Neutrophils for Figures 1–2 and 4–5. Neutrophils were considered CD11b+SiglecF‐Ly6G+ A. Neutrophils were identified initially by CD11b and Ly6G expression. These cells were confirmed to be SiglecF‐(B).

**Figure S4:** Brasilonema sp. LPS induces pro‐inflammatory cytokine production. C57Bl/6 mice were exposed to either PBS or Brasilonema sp. LPS for 24 hours before BALF was collected and analyzed using Milliplex analysis. Data are a combination of 3 mice and * indicates *p* < 0.05.

**Figure S5:** Brasilonema sp. LPS does not induce cell death in A549 cells either alone or with multiple concentrations of SPA‐4 peptide. A549 lung epithelial cell line were exposed to Brasilonema sp. LPS in the absence or presence of the TLR4 antagonist (SPA‐4) for 24 hours before supernatants were pulled for LDH analysis (Millipore‐Sigma, Burlington, MA, USA) following the manufacturers instructions.

**Figure S6:** Brasilonema sp. LPS requires TLR4 to induces pro‐inflammatory cytokine production in vivo. Wildtype (C57Bl/6) or TLR4‐deficient mice were exposed to either PBS or Brasilonema sp. LPS for 24 hours before BALF was collected and analyzed using Milliplex analysis. Data are a combination of 3–6 mice and * indicates *p* < 0.05.

## Data Availability

The data that support the findings of this study are available from the corresponding author upon reasonable request.

## References

[1] A. Ray and J. K. Kolls , “Neutrophilic Inflammation in Asthma and Association With Disease Severity,” Trends in Immunology 38 (2017): 942–954. 10.1016/j.it.2017.07.003 PMC5711587 28784414

[2] S. L. Taylor , L. E. X. Leong , J. M. Choo , et al., “Inflammatory Phenotypes in Patients With Severe Asthma are Associated With Distinct Airway Microbiology,” Journal of Allergy and Clinical Immunology 141 (2018): 94–103.e15. 10.1016/j.jaci.2017.03.044 28479329

[3] I. Ogulur , Y. Mitamura , D. Yazici , et al., “Type 2 Immunity in Allergic Diseases,” Cellular and Molecular Immunology 22 (2025): 211–242. 10.1038/s41423-025-01261-2 PMC11868591 39962262

[4] S. H. Cho , “Pharmacogenomic Approaches to Asthma Treatment,” Allergy, Asthma and Immunology Research 2 (2010): 177–182. 10.4168/aair.2010.2.3.177 PMC2892049 20592916

[5] C. L. Marshall , K. Hasani , and N. Mookherjee , “Immunobiology of Steroid‐Unresponsive Severe Asthma,” Frontiers in Allergy 2 (2021): 718267, 10.3389/falgy.2021.718267. PMC8974815 35387021

[6] Y. Matsumura , “Heterogeneity of Glucocorticoid Resistance in Patients With Bronchial Asthma,” International Journal of Biomedical Science 6 (2010): 158–166. PMC3615268 23675190

[7] R. Wadhwa , K. Dua , I. M. Adcock , J. C. Horvat , R. Y. Kim , and P. M. Hansbro , “Cellular Mechanisms Underlying Steroid‐Resistant Asthma,” European Respiratory Review 28 (2019): 190096. 10.1183/16000617.0096-2019 PMC9488801 31636089

[8] N. Aoi , T. Fuchiwaki , I. Morikura , H. Kawauchi , and T. Sakamoto , “Nasal Administration of Lipopolysaccharide Exacerbates Allergic Rhinitis Through Th2 Cytokine Production From Mast Cells,” Allergies 1 (2021): 216–224.

[9] P. G. Fallon and C. Schwartz , “The High and Lows of Type 2 Asthma and Mouse Models,” Journal of Allergy and Clinical Immunology 145 (2020): 496–498. 10.1016/j.jaci.2019.11.031 31812572

[10] D. Murakami , H. Yamada , T. Yajima , A. Masuda , S. Komune , and Y. Yoshikai , “Lipopolysaccharide Inhalation Exacerbates Allergic Airway Inflammation by Activating Mast Cells and Promoting Th2 Responses,” Clinical and Experimental Allergy 37 (2007): 339–347. 10.1111/j.1365-2222.2006.02633.x 17359384

[11] W. Yamada , S. Tasaka , H. Koh , et al., “Role of Toll‐Like Receptor 4 in Acute Neutrophilic Lung Inflammation Induced by Intratracheal Bacterial Products in Mice,” Journal of Inflammation Research 1 (2008): 1–10. 10.2147/jir.s3771 PMC3218721 22096342

[12] J. A. Swartzendruber , R. M. Del Toro , R. Incrocci , et al., “Lipopolysaccharide From the Cyanobacterium Geitlerinema sp. Induces Neutrophil Infiltration and Lung Inflammation,” Toxins 14 (2022): 267. 10.3390/toxins14040267 PMC9024439 35448876

[13] K. Wisniewska , A. U. Lewandowska , and S. Sliwinska‐Wilczewska , “The Importance of Cyanobacteria and Microalgae Present in Aerosols to Human Health and the Environment ‐ Review Study,” Environment International 131 (2019): 104964. 10.1016/j.envint.2019.104964 31351382

[14] Q. L. Yu and Z. Chen , “Establishment of Different Experimental Asthma Models in Mice,” Experimental and Therapeutic Medicine 15 (2018): 2492–2498. 10.3892/etm.2018.5721 PMC5795809 29456654

[15] M. Mehdizadeh Allaf and H. Peerhossaini , “Cyanobacteria: Model Microorganisms and Beyond,” Microorganisms 10 (2022): 696. 10.3390/microorganisms10040696 PMC9025173 35456747

[16] I. Stewart , P. J. Schluter , and G. R. Shaw , “Cyanobacterial Lipopolysaccharides and Human Health ‐ A Review,” Environmental Health 5 (2006): 7. 10.1186/1476-069X-5-7 PMC1489932 16563160

[17] M. Barbosa , D. E. Berthold , F. W. Lefler , and H. D. Laughinghouse , “Diversity of the Genus Brasilonema (Nostocales, Cyanobacteria) in Plant Nurseries of Central Florida (USA) With the Description of Three New Species: B. Fioreae Sp. nov., B. Santannae sp. Nov. and B. Wernerae sp. nov,” Fottea 21 (2021): 82–99.

[18] H. Annadotter , G. Cronberg , R. Nystrand , and R. Rylander , “Endotoxins From Cyanobacteria and Gram‐Negative Bacteria as the Cause of an Acute Influenza‐Like Reaction After Inhalation of Aerosols,” EcoHealth 2 (2005): 209–221.

[19] L. Backer , D. Manassaram‐Baptiste , R. LePrell , and B. Bolton , “Cyanobacteria and Algae Blooms: Review of Health and Environmental Data From the Harmful Algal Bloom‐Related Illness Surveillance System (HABISS) 2007‐2011,” Toxins 7 (2015): 1048–1064. 10.3390/toxins7041048 PMC4417954 25826054

[20] D. N. Facciponte , M. W. Bough , D. Seidler , et al., “Identifying Aerosolized Cyanobacteria in the Human Respiratory Tract: A Proposed Mechanism for Cyanotoxin‐Associated Diseases,” Science of the Total Environment 645 (2018): 1003–1013. 10.1016/j.scitotenv.2018.07.226 PMC6159226 30248825

[21] H. E. Plaas and H. W. Paerl , “Toxic Cyanobacteria: A Growing Threat to Water and Air Quality,” Environmental Science and Technology 55 (2021): 44–64. 10.1021/acs.est.0c06653 33334098

[22] X. Fan , P. Shu , Y. Wang , N. Ji , and D. Zhang , “Interactions Between Neutrophils and T‐Helper 17 Cells,” Frontiers in Immunology 14 (2023): 1279837. 10.3389/fimmu.2023.1279837 PMC10619153 37920459

[23] A. Frey , L. P. Lunding , and M. Wegmann , “The Dual Role of the Airway Epithelium in Asthma: Active Barrier and Regulator of Inflammation,” Cells 12 (2023): 2208. 10.3390/cells12182208 PMC10526792 37759430

[24] P. J. Barnes and I. M. Adcock , “Glucocorticoid Resistance in Inflammatory Diseases,” Lancet 373 (2009): 1905–1917. 10.1016/S0140-6736(09)60326-3 19482216

[25] C. Nunes , A. M. Pereira , and M. Morais‐Almeida , “Asthma Costs and Social Impact,” Asthma Research and Practice 3, no. 1 (2017): 1. 10.1186/s40733-016-0029-3 PMC5219738 28078100

[26] W. Wang , J. Jing Li , P. S. Foster , P. M. Hansbro , and M. Yang , “Potential Therapeutic Targets for Steroid‐Resistant Asthma,” Current Drug Targets 11 (2010): 957–970. 10.2174/138945010791591412 20412045

[27] R. A. Hughes , Y. Zhang , R. Zhang , P. G. Williams , J. S. Lindsey , and E. S. Miller , “Genome Sequence and Composition of a Tolyporphin‐Producing Cyanobacterium‐Microbial Community,” Applied and Environmental Microbiology 83, no. 19 (2017): e01068‐17. 10.1128/AEM.01068-17 PMC5601333 28754701

[28] M. Swanson‐Mungerson , R. Incrocci , V. Subramaniam , P. Williams , M. L. Hall , and A. M. S. Mayer , “Effects of Cyanobacteria Oscillatoria sp. Lipopolysaccharide on B Cell Activation and Toll‐Like Receptor 4 Signaling,” Toxicology Letters 275 (2017): 101–107. 10.1016/j.toxlet.2017.05.013 PMC5510639 28499610

[29] M. Swanson‐Mungerson , P. Williams , J. R. Gurr , et al., “Biochemical and Functional Analysis of Cyanobacterium Geitlerinema sp. LPS on Human Monocytes,” Toxicological Sciences 171, no. 2 (2019): 421–430. 10.1093/toxsci/kfz153 PMC6760288 31271425

[30] J. Papageorgiou , T. A. Linke , C. Kapralos , B. C. Nicholson , and D. A. Steffensen , “Extraction of Cyanobacterial Endotoxin,” Environmental Toxicology 19 (2004): 82–87. 10.1002/tox.10152 14758595

[31] B. O. Westphal and K. Jann , “Extraction with phenol‐water and further applications of the procedure.” Methods in Carbohydrate Chemistry (Academic Press, 1965).

[32] J. A. Swartzendruber , R. W. Incrocci , S. A. Wolf , A. Jung , and K. L. Knight , “Bacillus Subtilis Exopolysaccharide Prevents Allergic Eosinophilia,” Allergy 74 (2019): 819–821. 10.1111/all.13674 PMC7064269 30456900

[33] S. Hadjigol , K. G. Netto , S. Maltby , et al., “Lipopolysaccharide Induces Steroid‐Resistant Exacerbations in a Mouse Model of Allergic Airway Disease Collectively Through IL‐13 and Pulmonary Macrophage Activation,” Clinical and Experimental Allergy 50 (2020): 82–94. 10.1111/cea.13505 31579973

[34] S. Awasthi , N. Rahman , B. Rui , et al., “Lung and General Health Effects of Toll‐Like receptor‐4 (TLR4)‐interacting SPA4 Peptide,” BMC Pulmonary Medicine 20 (2020): 179. 10.1186/s12890-020-01187-7 PMC7310322 32576172

[35] D. C. Zeldin , C. Wohlford‐Lenane , P. Chulada , et al., “Airway Inflammation and Responsiveness in Prostaglandin H Synthase‐Deficient Mice Exposed to Bacterial Lipopolysaccharide,” American Journal of Respiratory Cell and Molecular Biology 25 (2001): 457–465. 10.1165/ajrcmb.25.4.4505 11694451

[36] K. A. Wiśniewska , S. Śliwińska‐Wilczewska , and A. U. Lewandowska , “The First Characterization of Airborne Cyanobacteria and Microalgae in the Adriatic Sea Region,” PLoS One 15 (2020): e0238808. 10.1371/journal.pone.0238808 PMC7482968 32913356

[37] L. Mazgaeen and P. Gurung , “Recent Advances in Lipopolysaccharide Recognition Systems,” International Journal of Molecular Sciences 21 (2020): 379. 10.3390/ijms21020379 PMC7013859 31936182

[38] I. M. Lorenzo , W. Liedtke , M. J. Sanderson , and M. A. Valverde , “TRPV4 Channel Participates in Receptor‐Operated Calcium Entry and Ciliary Beat Frequency Regulation in Mouse Airway Epithelial Cells,” Proceedings of the National Academy of Sciences 105 (2008): 12611–12616. 10.1073/pnas.0803970105 PMC2527959 18719094

[39] Y. S. Yi , “Caspase‐11 Non‐Canonical Inflammasome: A Critical Sensor of Intracellular Lipopolysaccharide in Macrophage‐Mediated Inflammatory Responses,” Immunology 152 (2017): 207–217. 10.1111/imm.12787 PMC5588777 28695629

[40] P. Raptova , V. Skockova , P. Babica , et al., “Cyanobacterial Bloom‐Associated Lipopolysaccharides Induce Pro‐Inflammatory Processes in Keratinocytes In Vitro,” Environmental Toxicology and Pharmacology 105 (2024): 104342. 10.1016/j.etap.2023.104342 38092246

[41] V. Skočková , P. Raptová , K. Pospíchalová , et al., “Cyanobacterial Harmful Bloom Lipopolysaccharides: Pro‐Inflammatory Effects on Epithelial and Immune Cells In Vitro,” Archives of Toxicology 98 (2024): 481–491. 10.1007/s00204-023-03644-8 PMC10794361 38063875

[42] V. Skočková , O. Vašíček , E. Sychrová , I. Sovadinová , P. Babica , and L. Šindlerová , “Cyanobacterial Harmful Bloom Lipopolysaccharides Induce Pro‐Inflammatory Effects in Immune and Intestinal Epithelial Cells In Vitro,” Toxins (Basel) 15, no. 3 (2023): 169, 10.3390/toxins15030169. PMC10058507 36977060

[43] A. Omidi , S. Pflugmacher , A. Kaplan , Y. J. Kim , and M. Esterhuizen , “Reviewing Interspecies Interactions as a Driving Force Affecting the Community Structure in Lakes via Cyanotoxins,” Microorganisms 9 (2021): 1583. 10.3390/microorganisms9081583 PMC8401979 34442662

[44] H. W. Paerl and J. Huisman , “Blooms Like it Hot,” Science 320 (2008): 57–58. 10.1126/science.1155398 18388279

[45] Z. Wang , S. Akbar , Y. Sun , et al., “Cyanobacterial Dominance and Succession: Factors, Mechanisms, Predictions, and Managements,” Journal of Environmental Management 297 (2021): 113281. 10.1016/j.jenvman.2021.113281 34274765

[46] H. Menden , E. Tate , N. Hogg , and V. Sampath , “LPS‐Mediated Endothelial Activation in Pulmonary Endothelial Cells: Role of Nox2‐dependent IKK‐Beta Phosphorylation,” American Journal of Physiology‐Lung Cellular and Molecular Physiology 304 (2013): L445–L455. 10.1152/ajplung.00261.2012 PMC3602745 23333803

[47] M. Wang , J. Feng , D. Zhou , and J. Wang , “Bacterial Lipopolysaccharide‐Induced Endothelial Activation and Dysfunction: A New Predictive and Therapeutic Paradigm for Sepsis,” European Journal of Medical Research 28 (2023): 339. 10.1186/s40001-023-01301-5 PMC10498524 37700349

[48] H. C. Müller‐Redetzky , N. Suttorp , and M. Witzenrath , “Dynamics of Pulmonary Endothelial Barrier Function in Acute Inflammation: Mechanisms and Therapeutic Perspectives,” Cell and Tissue Research 355 (2014): 657–673. 10.1007/s00441-014-1821-0 PMC7102256 24599335

[49] M. Molteni , A. Bosi , and C. Rossetti , “The Effect of Cyanobacterial LPS Antagonist (CyP) on Cytokines and Micro‐RNA Expression Induced by Porphyromonas Gingivalis LPS,” Toxins 10 (2018): 290. 10.3390/toxins10070290 PMC6071223 30012943

[50] A. M. S. Mayer , J. Murphy , D. MacAdam , et al., “Classical and Alternative Activation of Cyanobacterium Oscillatoria sp. Lipopolysaccharide‐Treated Rat Microglia In Vitro,” Toxicological Sciences 149 (2016): 484–495. 10.1093/toxsci/kfv251 PMC4900220 26609141

[51] Y. Yang , Z. Gao , M. Lou , J. Ma , and X. Li , “Hepatopancreatic and Intestinal Inflammation is Induced by Lipopolysaccharide From Microcystis in Common Carp via the TLRs/NF‐KappaB Pathway,” Fish and Shellfish Immunology 165 (2025): 110497. 10.1016/j.fsi.2025.110497 40518031

[52] S. Awasthi , G. Kumar , V. Ramani , et al., “Mechanism of Anti‐Inflammatory Activity of TLR4‐Interacting SPA4 Peptide,” Immunohorizons 5 (2021): 659–674. 10.4049/immunohorizons.2100067 PMC8673433 34429343

